# Pathophysiological Mechanisms of Severe Anaemia in Malawian Children

**DOI:** 10.1371/journal.pone.0012589

**Published:** 2010-09-07

**Authors:** Michaël Boele van Hensbroek, Job C. J. Calis, Kamija S. Phiri, Raymond Vet, Francis Munthali, Rob Kraaijenhagen, Henk van den Berg, Brian Faragher, Imelda Bates, Malcolm E. Molyneux

**Affiliations:** 1 Malawi-Liverpool-Wellcome Trust Clinical Research Programme, College of Medicine, University of Malawi, Blantyre, Malawi; 2 Emma Children's Hospital, Academic Medical Centre, Amsterdam, The Netherlands; 3 Liverpool School of Tropical Medicine, Liverpool, United Kingdom; 4 Department of Specialized Hematology, Academic Medical Centre, Amsterdam, The Netherlands; 5 Department of Clinical Chemistry, Meander Medical Center, Amersfoort, The Netherlands; National Institute of Child Health and Human Development, United States of America

## Abstract

**Background:**

Severe anaemia is a major cause of morbidity and mortality in African children. The aetiology is multi-factorial, but interventions have often targeted only one or a few causal factors, with limited success.

**Methods and Findings:**

We assessed the contribution of different pathophysiological mechanisms (red cell production failure [RCPF], haemolysis and blood loss) to severe anaemia in Malawian children in whom etiological factors have been described previously. More complex associations between etiological factors and the mechanisms were explored using structural equation modelling. In 235 children with severe anaemia (haemoglobin<3.2 mMol/L [5.0 g/dl]) studied, RCPF, haemolysis and blood loss were found in 48.1%, 21.7% and 6.9%, respectively. The RCPF figure increased to 86% when a less stringent definition of RCPF was applied. RCPF was the most common mechanism in each of the major etiological subgroups (39.7–59.7%). Multiple aetiologies were common in children with severe anaemia. In the final model, nutritional and infectious factors, including malaria, were directly or indirectly associated with RCPF, but not with haemolysis.

**Conclusion:**

RCPF was the most common pathway leading to severe anaemia, from a variety of etiological factors, often found in combination. Unlike haemolysis or blood loss, RCPF is a defect that is likely to persist to a significant degree unless all of its contributing aetiologies are corrected. This provides a further explanation for the limited success of the single factor interventions that have commonly been applied to the prevention or treatment of severe anaemia. Our findings underline the need for a package of measures directed against all of the local aetiologies of this often fatal paediatric syndrome.

## Introduction

Severe anaemia (haemoglobin concentration <3.2 mMol/L [<5.0 g/dl]) is a common cause of morbidity and mortality in African children [1]–[4]. Of all children admitted to hospital, 12–29% receive a blood transfusion and in-hospital mortality in this group is commonly between 4 and 10% [3], [5], [6].

The pathogenesis of anaemia is complex because several distinct *mechanisms* may lead to a reduced number of circulating red cells. In African children, mechanisms known to contribute to severe anaemia include haemolysis, acute or chronic blood loss, and red cell production failure (RCPF) [7]. Each of these mechanisms may be activated by a variety of *etiological factors* and some single aetiologies may affect more than one mechanism [4], [8]. For example, malnutrition, HIV and malaria may each cause RCPF, while a malaria infection may cause both haemolysis and RCPF. A single etiological factor may predominate in some patients, while in others multiple etiological factors and mechanisms may combine to result in severe anaemia [9].

Despite the size of the problem, severe anaemia has received little research attention and its pathophysiology is still poorly understood [10]. Interventional studies of either prevention or treatment of severe anaemia have often evaluated only one etiological factor at a time [7]. The limited success of this approach is not surprising if most of the severe anaemia in children is the result of multiple etiological factors as a recent study suggested [10]. Measures directed against only some of the aetiologies in an individual are particularly likely to fail if RCPF is a major pathogenetic pathway, since RCPF is unlikely to be reversed unless all of its contributing causes are corrected. By contrast, haemolysis and blood loss may be, at least partially, alleviated if some aetiologies are removed while some remain. In order to adopt a rational approach to reducing the burden of severe anaemia it is important to be able to identify both the important mechanisms and the specific etiological factors that contribute to these mechanisms.

To do so we studied a group of rural and urban Malawian children with severe anaemia. The aetiologies associated with anaemia in these children have been reported previously [10]. We now analyse the pathogenetic mechanisms of anaemia prevailing in the same children, and present a profile of the syndrome that describes both aetiologies and mechanistic pathways in children presenting with severe anaemia.

## Methods

### Population

This study formed part of a large severe anaemia research programme conducted in southern Malawi at Queen Elizabeth Central hospital in Blantyre (urban site) and Chikwawa District hospital (rural site). Between July 2002 and July 2004 consecutive children with a primary diagnosis of severe anaemia were recruited. The etiologic factors and agents associated with anaemia in these cases have been reported elsewhere [10].

Children were eligible for enrolment if their blood haemoglobin concentration was less than 3.2 mMol/L, they were aged 6–60 months and had not received a blood transfusion within the preceding month. Informed consent was obtained from a parent or guardian. The study was approved by the ethics committees of the College of Medicine, University of Malawi and the Liverpool School of Tropical Medicine, United Kingdom.

### Procedures

Procedures for admission and patient management have been described in detail elsewhere [10]. In summary, on enrolment a standardized study questionnaire and physical examination were completed, and samples of blood, urine and stool were collected. If the clinical condition permitted, a fine needle bone marrow aspirate was obtained under anaesthesia from either the posterior superior iliac spine or the anterior iliac crest. Bone marrows were obtained from 348 children (91.3%), the remaining 8.7% were too ill for the bone marrow aspirate procedure. Children were treated in a study ward and all conditions were managed according to standard protocols.

### Laboratory measurements

Laboratory tests, crucial to patient management, were performed within 24 hours, and sample aliquots were stored at −80°C for later analysis. Whole blood haemoglobin and plasma haemoglobin concentrations were measured using the HaemoCue system® (Angelholm, Sweden). A full blood count, including absolute reticulocyte count, was performed by Coulter counter analyzer® (Beckman Coulter, Durban, South Africa). Bone marrow aspirates were used for microscopic evaluation and for measurement of the red cell precursor fractions on a FACS-Calibur flow-cytometer (Becton/Dickinson Biosciences, San Jose,CA USA). Wedged spread films were air-dried, fixed in methanol and stained with May-Grüwald-Giemsa and for iron (HematoGnost Fe, Darmstadt, Germany). Bone marrow differential count was done by an experienced laboratory technician (RV). Iron stained bone marrow slides were graded according to Gale's criteria for iron content in stroma, macrophages and red cell precursors [11].

C-reactive protein, erythropoietin, haptoglobin, bilirubin, ferritin, folate and vitamin B12 concentrations were analyzed in heparin plasma on a Roche p800 system (Roche, Switzerland). Serum vitamin A (retinol) and soluble transferrin receptor (sTfR) were measured using high performance liquid chromatography and enzyme linked immunosorbant assay (Ramco Laboratories, TX) respectively [12]. *Plasmodium falciparum* asexual parasites were counted against 200 white blood cells and parasite density was calculated from the total white cell count. Stool samples were examined for helminths using the Kato-Katz method and polymerase chain reaction (PCR) [13]–[15].

A bone marrow or venous blood sample (1–2 ml) was inoculated into BACTEC Myco/F-Lytic culture vials and incubated in a BACTEC 9050 automated system for 56 days [16]. Cultures were checked for mycobacteria using Ziehl-Neelsen stained slides. Mixed growth or growth of micrococci, Bacillus species or coagulase-negative staphylococci were considered contaminants. HIV testing was performed using two rapid tests (Determine, Abbott-Laboratories, Japan; Unigold, Trinity-Biotech, Ireland); discordant results and positive tests in children less than 18 months being resolved by PCR.

### Definitions used

*Anaemia mechanisms:* The criteria used to define the three severe anaemia mechanisms are given in table 1.

**Table 1:** Pathophysiological mechanisms in Malawian children with severe anaemia.

Mechanism	Definitions used	Prevalence (n) #
**Red Cell Production Failure (RCPF)**	Whole blood haemoglobin <3.2 mMol/L (5.0 g/dL) and Reticulcytes <50,000/uL †	48.1% (113/235)
**Haemolysis**	Plasma haemoglobin >0.10 mMol/L (0.15 g/dl) and/or Bilirubin >16.9 mmol/L	21.7% (51/235)
**Blood loss**	UT: Urine dip-stick >1+ for erythrocytes and/or GI: Hookworm load >1000 eggs/gram stool *	7.2% (17/235)
**Non defined**	Not fulfilling any of the above criteria	34.5% (81/235)

GI  =  Gastro-intestinal, UT  =  Urinary Tract.

# Includes patients with a complete dataset only.

3*Gastro-intestinal (GI) blood loss was measured indirectly, using a hookworm infection as a substitute marker.

† If a reticulocyte cut-off <150,000/uL is used to define RCPF, 86.4% (203/235) of patients would fall into this category, with a 23.0% (54/235) overlap with haemolysis and blood loss.

*Etiological factors*: Important etiological factors in the study population were defined by multivariate analysis and included: malaria, HIV, bacteraemia, hookworm infection and iron, vitamin B12 and vitamin A deficiency [10].

*Dyserythropoiesis:* was defined by the following nuclear features in bone marrow smears: (a) multi-nuclearity; (b) karyorrhexis; (c) intercellular chromatin bridging; and (d) incomplete mitoses. Dyserythropoiesis was expressed quantitatively as the percentage of RBC precursors fulfilling at least one of these criteria^4^.

*Nucleated red cells* (NRC): were defined as all mononuclear bone marrow cells positively stained with LDS-751 (a DNA dye) and expressing CD235a (Glycoprotein A) on flow-cytometric analysis.

*Red cell production failure (RCPF):* was defined as a whole blood haemoglobin <3.2 mMol/L (5.0 g/dL) and Reticulocytes <50,000/uL.

*Malaria infection:* was defined as the presence of asexual *Plasmodium falciparum* (Pf) parasites on a blood film; Parasitaemia in excess of 10,000 asexual parasites/µL was referred to as ‘Pf>10^4^/ul’.

*Iron deficiency:* In our study population, a sTfR/log ferritin-index of more than 5.6 was used to define iron deficiency. This index (and cut off) has been shown to be the best predictor of bone marrow iron status irrespective of the presence of infection with a sensitivity of 70%, specificity of 75%) [17].

*Gastro-intestinal blood loss:* was not measured directly. A hookworm load of more then 1000 ova per gram of stool was used as substitute marker.

### Statistical Methods

This is primarily a study of the diversity of mechanisms of severe anaemia, the identification of which includes an assessment of bone marrow appearances. The primary statistical analysis is thus purely descriptive. The characteristics of the study cohort are summarized using frequency counts/percentages for categorical factors and mean values (with their standard deviations) for continuous factors. The prevalence of each of the four pathophysiological mechanisms is summarized using frequency counts/percentages, as are the markers and etiological factors for each of the mechanisms (with the single exception of the marker NRC which, because of its skewed continuous statistical distribution, is summarized using medians and inter-quartile ranges).

The associations between etiological factors and mechanisms were explored using Structural Equation Modelling (SEM). SEM is a complex but increasingly popular data-analytical technique, the strengths and limitations of which are well documented [18], [19]. Conventional regression methods were considered too simplistic to investigate the complex relationships hypothesized between the projected etiological factors and the mechanisms under investigation, for two reasons. Firstly, there were multiple mechanisms, so hence multiple outcome measures to be considered simultaneously (although ultimately only two mechanisms were evaluated together). Secondly, three of the etiological factors (“group 1”: hookworm, HIV and vitamin A deficiency) were not considered to be influencing the mechanisms directly, but only indirectly through relationships with the remaining four projected etiological factors (“group 2”: iron deficiency, vitamin B12 deficiency, bacteraemia and malaria); furthermore, the possibility that the three “group 1” factors could each be influencing different “group 2” factors needed to be explored, a complexity outside the scope of conventional multi-stage multiple regression models.

In the initial SEM model, all three “group1” etiological factors were considered to be associated with all four “stage 2” factors, and all four “stage 2” factors were considered to be associated with both mechanisms examined (red cell production failure and haemolysis). Relationships found to be statistically non-significant were removed by backward elimination. The arrows indicate the directions of the hypothesized relationships, statistically significant at the conventional alpha  = 0.05 level. The figures adjoining each arrow are the standardized regression coefficients from the SEM model; these indicate the (mathematical) strength of the relationships and can be interpreted similarly to conventional correlation coefficients. The goodness-of-fit of the final SEM was determined using the RMSEA (root mean square error of approximation) statistic – a value of RMSEA <0.05 indicates an close model fit and a value <0.08 indicates a reasonable model fit^18^.

All analyses were carried out using the STATA 9 (Stata Corporation, TX), SPSS 14 and AMOS 7.0 (SPSS, IL) statistical computer packages.

The study was approved by the ethics committees of the College of Medicine, University of Malawi and the Liverpool School of Tropical Medicine, United Kingdom.

## Results

**Figure 1:**
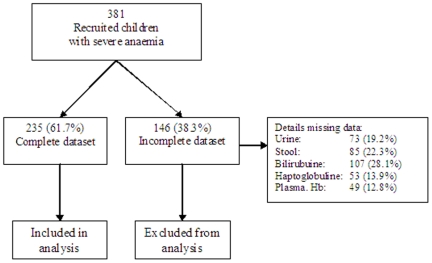
Flow chart showing from the number of children with severe anaemia recruited to the number of children analysed for the various severe anaemia mechanisms. The main reasons for missing data were: early death, insufficient sample and failed laboratory assays.

**Figure 2:**
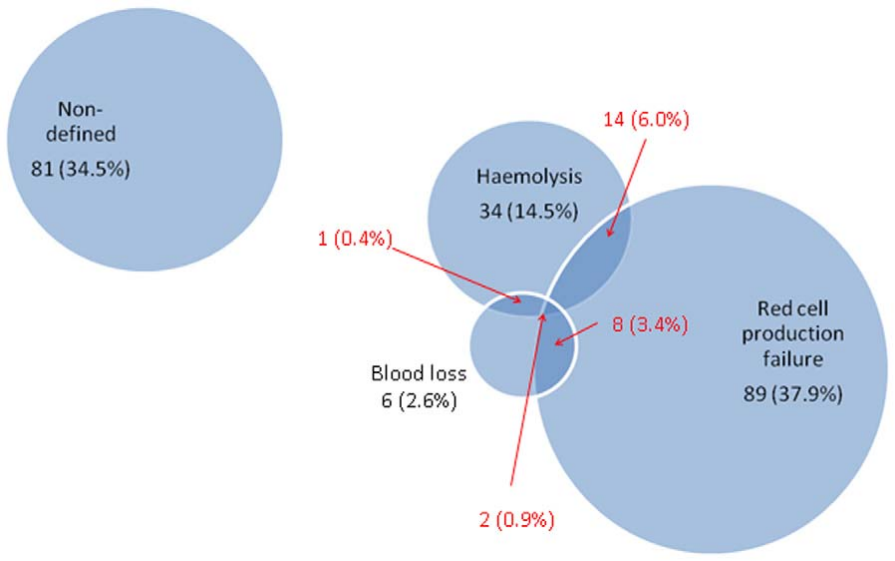
Pathophysiological mechanisms in Malawian children with severe anaemia syndrome. Non-defined  =  not fulfilling the definitions for red cell production failure, haemolysis or blood loss. Number and percentage given: mechanism sub-groups not overlapping (black) and overlapping (red).

Three-hundred-and-eighty-one children with severe anaemia (patients), 205 (53.8%) from Blantyre (urban) and 176 (46.2%) from Chikwawa (rural), were enrolled over a two year period (2002-04). The median age of the children was 16.5 (range 6-59.6) months and 46.7% (n = 178) were boys (table 2). Of these 381, 235 (62%) had a complete dataset for all the mechanism-defining variables (figure 1 and table 1) and were included in the main analysis. Incompleteness of the dataset was commonly caused by early death (not able to obtain urine and stool samples), insufficient sample volume or failure of one of the laboratory assays. Of the children with a complete dataset129 (54.9%), 23 (9.8%) and 2 (0.9%) fulfilled the definitions for one, two and three anaemia mechanisms, respectively (figure 2).

**Table 2:** Baseline variables of severely anaemic children.

	
	Included in all analyses	Excluded from analysis	P
Age in months, mean (SD)	19.9 (12.8)	21.1 (12.7)	0.4
Gender, boys, % (No)	48.1 (113/235)	44.5 (65/146)	0.5
Study site, rural, % (No)	49.8 (117/235)	40.4 (59/146)	0.1
Haemoglobin in g/dl, mean (SD)	3.6 (0.8)	3.6 (0.9)	0.7

### Mechanisms

Red cell production failure (RCPF) was identified in 113 (48.1%) of 235 children with severe anaemia. Of those with RCPF, 24 (21.3%) also had other mechanisms (figure 2). By a less stringent definition, using a reticulocyte cut-off of less then 150,000/uL (∼3% of erythrocytes in a non anaemic child [18]), 86% of all severely anaemic children fell into the RCPF category (table 1). The RCPF group was explored in more detail by determining the percentage of nucleated red cells (NRC) and by the presence of features of dyserythropoiesis. It was found that in the children with RCPF 11.4% (inter quartile range 4.3-36.9) of the mononuclear fraction was defined as NRC. Dyserythropoietic features in the NRC were found in 61.3% of the children with RCPF with a mean of 3.6% (standard deviation 2.0) of cells affected (table 3).

**Table 3:** Laboratory markers associated with severe anaemia mechanisms.

Markers	Mechanisms			
	RCPF % (n)	Haemolysis % (n)	Blood loss % (n)	Non-defined % (n)
**CRP** Elevated (>10 mmol/L)	85.3 (93/109)	89.4 (42/47)	64.7 (11/17)	93.8 (75/80)
**Erythropoietin** Not elevated (≤1200 U/L)	13.8 (8/58)	7.7 (2/26)	0.0 (0/6)	10.6 (5/47)
**Haptoglobin** Low (<0.2 mMol/L)	68.6 (72/105)	86.4 (38/44)	52.9 (9/17)	78.9 (60/76)
**Dyserythropoiesis** #	61.3 (49/80)	62.9 (22/35)	69.2 (9/13)	55.9 (33/59)
**NRC** Median % [IQR] *	11.4 [4.3-36.9] (85)	16.5 [4.9-41.9] (43)	26.1 [16.1-34.2] (14)	17.0 [7.7-38.9] (59)

# Defined by the presence of dyserythropoietic features in >2% of red cell precursors.

*Median [Inter Quartile Range] percentage Nucleated Red Cells (NRC) of all mononuclear bone marrow cells. Note that (n) may vary within the mechanism column due to missing values.

Haemolysis was found in 51 out of 235 patients (21.7%), of whom 17 (33.3%) also had criteria for RCPF and/or blood loss (figure 1). Of the 235 patients 19.3% had elevated plasma haemoglobin levels, indicative of intra-vascular haemolysis, and 5.0% had a raised bilirubin level. In the haemolysis group 24.4% (IQR 16.2-40.9) of the mononuclear fraction was defined as NRC and dyserythropoiesis was found in 62.9% of children with haemolysis.

Evidence of blood loss was found in 17 patients (7.2%): this was through the urinary tract in 5 (2.1%) and the gastro-intestinal tract in 12 (5.1%).

### Etiological factors

Among all previously identified important etiological factors of severe anaemia^10^, the most common etiological factors were found to be vitamin A deficiency, malaria infection, iron and vitamin B12 deficiency in 92.3, 59.5, 46.6 and 30.4 percent of patients, respectively [10]. RCPF was the most common associated mechanism, with 38.7 - 59.7% of patients, in the various etiological subgroups, fulfilling the definition of RCPF (table 4).

**Table 4:** Pathologic mechanism in Malawian children with severe anaemia by main etiological factors present on hospital admission.

Mechanisms	Etiological factors†										
	Infection								Nutrition		
	Malaria		HIV		Bacteraemia		Hook-worm		Iron status	Vitamin B12	Vitamin A
	Positive		Positive		Positive		Positive		Deficient	Deficient	Deficient
	all	No other infection	all	No other infection	all	No other infection	all	No other infection			
	145	87	31	7	35	13	23	12	73	62	148
**Red Cell Production Failure, % (n)**	42.1 (61)	40.2 (35)	38.7 (12)	85.7 (6)	48.6 (17)	30.8 (4)	52.2 (12)	66.7 (8)	50.7 (37)	59.7 (37)	44.6 (66)
**Haemolysis, % (n)**	17.9 (26)	17.2 (15)	19.4 (6)	28.6 (2)	25.7 (9)	38.5 (5)	13.0 (3)	8.3 (1)	13.7 (10)	22.6 (14)	22.3 (33)
**Non Defined, % (n)**	41.4 (60)	43.7 (38)	48.4 (15)	14.3 (1)	25.7 (9)	30.8 (4)	26.1 (6)	25.0 (3)	37.0 (27)	24.2 (15)	39.2 (58)

‘all’  =  all children with the indicated infection as a single infection or part of multiple infections ‘No other infection’  =  Children with the indicated infection only (as single infection).

† Folate deficiency (<3.0 µg/L) was not found and therefore not included in the table. Concentrations of vitamin B12<200 ng/L and vitamin A<20 µg/dL were considered deficient. Note that discrepancies between total numbers given (in column top number) and the sum of mechanisms in the respective column are due to overlap and excluding the ‘blood loss’ group (blood loss group was excluded because only a small number (n = 17) fulfilled the definition).

Infections (viral/bacterial/parasitic) associated with anaemia occurred in 211/275 (76.4%) of the severe anaemia patients evaluated of whom 77.7% (n = 164) had one infection, an additional 18.0% (38) had two and 8.5% (9) three infections. A single infection (isolated) was relatively uncommon (22.6%) among the HIV infected patients, but common among the malaria, hookworm and bacteraemia infected patients, in whom 60.0, 52.2 and 37.1% were isolated infection, respectively (table 4).

RCPF was also the most important mechanism in the sub-groups of children with a single infection. Only in the six children with bacteraemia was haemolysis more commonly found. In the children with a positive malaria slide, RCPF and haemolysis were found in 61 (42.1%) and 26 (17.9%) and in the sub-group with a high malaria parasite count (more then 10^4^/uL), the equivalent proportions were 36.6% and 17.1%.

### Structural Equation Model

**Figure 3:**
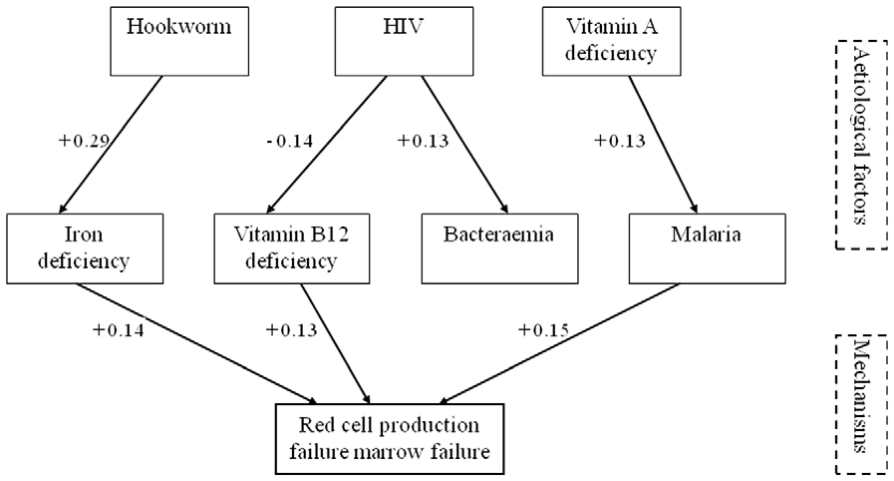
Structural Equation Model. Exploratory model describing the relationship between the important etiological factors and the mechanisms: Red Cell Production Failure and Haemolysis. A reduced model is presented, in which the significant (→)relationships are indicated (non-significant arrows are taken out for clarity). Size of the associations is indicated by numbers (standardized regression coefficients; range: -1.0/+1.0). The overall model fit was adequate (RMSEA: 0.055 (0.039-0.072)).

A structural equation model was developed in order to further explore the association between the anaemia mechanisms and etiological factors and to correct for interaction between factors ^17^. In the model, with a goodness of fit (RMSEA) of 0.053 (0.038-0.068), iron deficiency, vitamin B12 deficiency and malaria were directly and significantly associated with RCPF (standard regression coefficient of +0.14, -0.13, +0.15, respectively). Associations between RCPF and HIV, hookworm and vitamin A deficiency were indirect, through the iron and vitamin B12 deficiency and malaria respectively. No significant associations were found between the etiological factors and haemolysis (figure 3).

## Discussion

Severe anaemia in children in Africa has usually been investigated in the context of a condition that may contribute to it, such as malaria, malnutrition or helminth infection. We have studied the syndrome of severe anaemia in its own right, by enrolling consecutive Malawian children presenting to hospital with severe anaemia, irrespective of its possible aetiology. This has allowed us to build up a picture of contributory causes. To describe such causes, we have identified, for each child, both the mechanistic pathway leading to their anaemia (failure of red cell production, reduced red cell lifespan, or loss of red cells through haemorrhage) and the etiological factors that operated through these mechanisms (reported earlier [10]). These associations suggest, but they do not prove, a causal relationship between the etiological factors, the mechanisms and the development of severe anaemia. A description of severe anaemia from these two different perspectives provides a profile of the syndrome for the populations we have studied and a potential basis for the design of effective interventions.

Our results confirm that in Malawian children, although severe anaemia is associated with many potential etiological factors, one mechanism – failure of red cell production (RCPF) – predominates as the final pathway leading to anaemia. This was true when using stringent criteria for RCPF (reticulocytes <50,000/uL and haemoglobin <3.2 mMol/L [5 g/dl]) and became even more apparent when the cut-off for reticulocyte count was put at <150,000/uL (equal to 3% of erythrocytes in a non-anaemic child) [20], when 86% of all severely anaemic children fell into the RCPF category. The importance of RCPF may have been expected, since the study was conducted in an area with a high infection pressure, reflected in a high prevalence of single or mixed infections, and in the fact that the majority of patients had a raised plasma CRP concentration. In these circumstances, RCPF may be the result of pro-inflammatory cytokine activation giving rise to apoptosis of red cell precursors (nucleated red cells), dyserythropoiesis and down-regulation of erythropoietin production, with additional blunting of the effect of erythropoietin on the bone marrow (7). This interpretation – that severe anaemia is largely mediated by inflammatory mechanisms – is in part supported by the results listed in Table 3. However, inflammatory responses may not fully explain the RCPF, since nearly a quarter (23.6%) of children with stringently-defined RCPF were not found to have an infectious aetiology, while nutritional deficiencies, which are on their own able to directly affect red cell production, were found in over 40% of RCPF patients.

The dominance of RCPF was found in all etiological sub-groups, with surprisingly little variation between the etiological factors. Of additional importance was the observation that in the majority of children with severe anaemia multiple aetiologies (nutritional deficiencies and single or, in one fifth of patients, multiple infections) were found, each with the potential to cause a RCPF on its own. In such circumstances, RCPF may not be corrected or even ameliorated unless all of its contributing aetiologies are removed. This finding may explain why interventions directed against one or two aetiologies only to treat or prevent severe anaemia may be limited in their success [21]. It may also be an additional explanation of the high post-discharge mortality rate found in Kenyan children following a severe anaemia-with-Parasitaemia episode treated with blood transfusion and antimalarial drugs only [3].

The Structural Equation Model underlines both the importance of RCPF and the complexity of the syndrome, and indicates the apparently minor role of haemolysis in the patients we studied. There were no significant associations between haemolysis and any of the aetiologies, including malaria. This was the case whether malaria was defined as any Parasitaemia or a parasite density more then 10^4^/l. In many respects, it is the relationships that were found to be non-significant and hence removed from the SEM that justify the use of this complex mathematical methodology in this context. With conventional multi-stage regression models, all three of the “stage1” etiological factors (hookworm, HIV and vitamin A deficiency) would have to be assumed to be influencing (in some way) all four of the “stage 2” factors (iron deficiency, vitamin B12 deficiency, bacteraemia and malaria). However, the SEM shows clearly that this is not the case. Furthermore, the strengths of the relationships were estimated taking into account the possibility that etiological factors can influence more than one mechanism simultaneously, which is not easily done using conventional regression methods [19].

A limitation of a study of this kind is that data can be collected at one time point only, in a disease process that may have lasted for a long time with markers fluctuating from day to day. We have tried to limit this effect by using a large sample size. Another limitation is the difficulty of quantifying haemolysis and gastro-intestinal blood loss. The kinetics of free haemoglobin in plasma are not well known, and 1 g/dl of free haemoglobin is sufficient to saturate circulating haptoglobin, limiting the value of haptoglobin as quantitative marker of haemolysis [22]. For gastro-intestinal blood loss we assumed that hookworm is the only likely cause in these Malawian children and that blood loss is likely to accompany a heavy egg-load in the stool^8^.

These limitations may, in theory have caused some underestimation of blood loss and overestimation of haemolysis. However, they are unlikely to have affected the main findings, importance of RCPF, of our study and their implications for the control of severe anaemia in Malawi and similar settings. The syndromic approach that we have adopted allows a profile of paediatric severe anaemia to be developed, based on a description of both aetiologies and mechanisms for a particular population. In the case of the population we have studied, this profile indicates that although aetiologies are often multiple, the final pathogenetic pathway leading to severe anaemia is most commonly bone marrow failure. Any one of the aetiologies may on its own be sufficient to sustain this failure of red cell production. The next step will be to confirm our findings in other settings and to investigate causality between etiological factors, proposed mechanisms and severe anaemia. This may be followed by the design of a package of preventive and therapeutic measures (which may vary between different settings in Africa), aimed at reducing the burden of this common, often fatal, but neglected syndrome.
